# Introduction of a pilot program to measure and improve the clinical care of melanoma patients in the Lower Silesian Voivodeship in Poland: a report of 20 months experience

**DOI:** 10.1186/s12885-022-10253-8

**Published:** 2022-11-23

**Authors:** Marcin Ziętek, Jędrzej Wierzbicki, Edyta Pawlak, Adam Maciejczyk, Rafał Matkowski

**Affiliations:** 1 Department of Oncology, Wrocław Medical University, 12 Hirszfeld Square, 53-413 Wrocław, Poland; 2 Dolnośląskie Centrum Onkologii, Pulmonologii i Hematologii (Lower Silesian Oncology, Pulmonology and Hematology Center), 12 Hirszfeld Square, 53-413 Wrocław, Poland; 3 Laboratory of Immunopathology, Department of Experimental Therapy, Hirszfeld Institute of Immunology & Experimental Therapy, Polish Academy of Sciences, Rudolf Weigl 12 Street, 53-413 Wroclaw, Poland

**Keywords:** Melanoma, Pilot study, Benchmarking, Quality indicators, Patient pathway

## Abstract

**Background:**

In recent years, benchmarking and assessment methods to improve the quality of care have become increasingly important. Such approaches allow for a uniform assessment, comparisons between centers or over time, and the identification of weaknesses. In this study, the results of a 20-month pilot program to assess, monitor and improve the quality of care in melanoma patients primarily treated surgically are presented.

**Methods:**

The pilot program started in May 2020 at the Lower Silesian Oncology, Pulmonology and Hematology Center (LSOPHC) in Wroclaw, Poland (Lower Silesian Voivodeship, southwestern province of Poland with a population of 2,9 million). The program involved the introduction of a synoptic histopathological protocol, medical coordinators, and a set of measures to assess oncological care. In total, 11 Skin Cancer Unit (SCU) measures were introduced to analyze clinical outcomes, diagnostic quality, and duration. Data from 352 patients covered by the program were analyzed. In addition, the completeness of diagnostics from external sites was compared to our own results. Furthermore, the timeliness of the initial diagnostic tests and in-depth diagnostics were assessed and compared to the timeliness before implementation of the pilot program.

**Results:**

The introduced measures assessed the mortality related to oncological treatment, the rate of complications, advanced stages of melanoma, the completeness and duration of diagnostics, the involved nodes after lymphadenectomy, and melanoma screening. During the study period, the timeliness of the initial diagnostics was maintained at 87.8%, and the timeliness of the in-depth diagnostics at 89.5%. Compared to a similar period before the program, these values were 36.1% and 67.5%, respectively.

**Conclusion:**

The introduced measures seem to be effective and practical tools for benchmarking clinical and diagnostic aspects. They also allowed for a sensitive assessment of individual issues and indicated sensitive points. Furthermore, the actions undertaken in this pilot program allowed for a shortening of the duration of diagnostics.

## Introduction

The management of oncological patients is one of the major challenges for the healthcare system. Due to in part to the health and social importance of this disease, great emphasis is placed on optimizing the quality of treatment [1]. The increasing incidence of cancer, the necessity to provide a wide range of tests and therapeutic options in line with current guidelines, and the steadily growing costs of novel treatments have increased the importance of refining current oncological care [2, 3].

In Poland, the National Cancer Network’s Pilot Program (NCNPP) covering patients with lung, breast, colorectal, prostate and ovarian malignancies was introduced in 2019 to analyze and improve the quality of oncological treatment [4]. This program was implemented at selected centers and one of the main strategic goals was to improve oncological care in relation to the other European countries [5]. As part of the NCNPP, several organizational changes were carried out, including the creation of an oncology hotline, which enabled patients to sign up for diagnostic tests and obtain basic information about oncological treatment [5]. Moreover, quality measures and indicators were created to consistently assess the quality of services in units where the program was conducted [6, 7].

The neoplasms to which attention was directed under the NCNPP are the most commonly diagnosed worldwide and have a high mortality rate [8]. However, among newly diagnosed oncological patients, people with melanoma constitute a relatively large group, which, in Poland, includes 3,000 cases a year [9, 10]. Furthermore, the incidence of melanoma is steadily increasing (doubling every decade) according to recent global studies [11, 12].

One of the provinces where the NCNPP was implemented was the Lower Silesian Voivodeship located in south-west Poland, an area inhabited by 2.88 million people (data from the Polish Central Statistical Office for 2021). In this region, more than 250 melanomas are diagnosed annually. Thus, due to the aforementioned factors, an independent pilot program for melanoma was designed and introduced, and the results are presented here. The main objective of this study was to analyze the results of a 20-month pilot program implemented to assess, and consequently improve the quality of selected aspects of oncological care in patients with melanoma.

## Materials and methods

The pilot program presented in this study was implemented at the Lower Silesian Oncology, Pulmonology and Hematology Center (LSOPHC) in Wroclaw (Lower Silesian Voivodeship, Poland). This program covered patients in whom surgery was the primary treatment due to newly diagnosed melanoma, local recurrence or in-transit metastasis. The project started on May 1, 2020 and is ongoing. The activities undertaken in the project included:

- standardizing the results of pathomorphological examinations.
- assessing the quality of pathomorphological examinations both at the LSOPHC and at external sites.
- introducing the role of a medical coordinator.
- introducing measures evaluating selected factors of oncological care and further analysis of collected data.
- examination of data on the duration of diagnostics and its timeliness in the context of the Polish public health care system.

Introduced changes in hospital functioning were aimed at improving the quality of care and the prepared measures were used to assess the condition and identify weak points which later was supposed to allow for the improvement of individual aspects.

A previously developed synoptic histopathological protocol (HP) was introduced at the LSOPHC at the start of the pilot program. The protocol was developed in accordance with national guidelines and the College of American Pathologists protocol for melanoma [13, 14]. The protocol contains all of the currently recommended parameters for assessing the advancement of the primary melanoma (i.e., neuroinvasion, immunohistochemistry), as well as metastasis to the lymph nodes (i.e., capsular invasion).

Another step taken was the introduction of medical coordinators, whose duties included general support for the patient, informing the patients and their families about ongoing treatment, and planning the dates for diagnostic tests and visits to the clinic. The medical coordinators were also responsible for managing the patient’s DILO system (pol. system Diagnostyki i Leczenia Onkologicznego; eng. Diagnostic and Oncological Treatment system), which was introduced in Poland in 2015 to provide a fast-track pathway for patients with suspected cancer [15]. The system guarantees that the National Health Fund pays the costs of all procedures and that each patient will be diagnosed and receive the required treatment [16]. However, exceeding a predetermined deadline (3 weeks for initial diagnostics and 4 weeks for in-depth diagnostics) results in a reduction in financing from 100 to 70% [16].

In total, 11 benchmarking Skin Cancer Unit (SCU) measures were introduced at the start of pilot study (the methodology for calculating the measures is described in detail in Table 1). The F_1 SCU and F_4 SCU measures calculated the percentage of patient deaths relative to the time of diagnosis (number of deaths within 1 year of diagnosis) and treatment (number of deaths within 30 days following surgery, chemotherapy or radiotherapy). The F_5 SCU and F_6 SCU measures monitored complications requiring hospitalization or additional medical advice (consultation or outpatient assistance) in the 30-day postoperative period. During the pilot study, the percentage of patients with stage III and IV melanoma was examined using the F_7 SCU measure. The F_8 SCU measure assessed the completeness of HP examinations at our own treatment center and at other sites, in relation to the introduced protocol. Completeness was assessed at the end of the diagnostics during the multidisciplinary tumor board meeting by using a previously prepared checklist. The F_9 SCU measure determined the average time between registration and obtaining the results of diagnostic examinations. Wait times for ultrasonography (USG), computed tomography (CT), positron emission tomography (PET), and HP examinations were determined. The percentage of involved nodes harvested during lymphadenectomy was assessed using the F_10 SCU measure, and the percentage of patients divided according to the thickness of the infiltration according to the Breslow scale was assessed using the F_11 SCU measure. Clinical data and data from the checklist were imported into designated software - MedStreamDesigner (Transition Technologies Science, Warsaw, Poland) and there, through query modeling, measure reports were created. Patients’ medical records and raw data were not used in this analysis, but only aggregate data derived from the pilot program.

**Table 1:** Descriptions of the Skin Cancer Unit (SCU) measures

Measure	Area	Description and formula
**F_1 SCU**	Clinical outcome	General mortality
		$$\text{F}_{1}=\frac{\text{number of deaths with in one year of diagnosis}}{\text{number of patients}}$$
**F_2 SCU**	Clinical outcome	Mortality after surgical treatment
		$$\text{F}_{2}=\frac{\text{number of deaths with in 30 days after surgery}}{\text{number of patients after surgery}}$$
**F_3 SCU**	Clinical outcome	Mortality after chemotherapy
		$$\text{F}_{3}=\frac{\text{number of deaths with in 30 days after last cycle of chemotherapy}}{\text{number of patients receiving chemotherapy}}$$
**F_4 SCU**	Clinical outcome	Mortality after radiotherapy
		$$\text{F}_{4}=\frac{\text{number of deaths with in 30 days after last cycle of radiotherapy}}{\text{number of patients receiving radiotherapy}}$$
**F_5.1 SCU**	Clinical outcome	Occurrence of major complications after surgical treatment
		$$\text{F}_{5.1}=\frac{\text{number of patients requiring hospitalization within 30 days after surgery}}{\text{number of patients after surgery}}$$
**F_5.2 SCU**	Clinical outcome	Occurrence of minor complications after surgical treatment
		$$\text{F}_{5.2}=\frac{\text{number of patients requiring additional medical advice with in 30 days after surgery}}{\text{number of patients after surgery}}$$
**F_6 SCU**	Clinical outcome	Occurrence of complications after radiotherapy
		$$\text{F}_{6}=\frac{\text{number of patients requiring hospitalization with in 30 days after radiotherapy}}{\text{number of patients receiving radiotherapy}}$$
**F_7 SCU**	Diagnosis	Distribution of stage III and IV melanoma patients
		$$\text{F}_{7}=\frac{\text{number of patients with stage III/IV melanoma}}{\text{number of patients}}$$
**F_8.1 SCU**	Diagnostics	Completeness of initial diagnostics
		$$\text{F}_{8.1}=\frac{\text{number of complete initial diagnostic tests}}{\text{number of initial diagnostic tests}}$$
**F_8.2 SCU**	Diagnostics	Completeness of in-depth diagnostics
		$${F}_{8.2}=\frac{numberofcompletein-depthdiagnostictests}{numberofin-depthdiagnostictests}$$
**F_9 SCU**	Diagnostics	Waiting time for diagnostic results
		$$\text{F}_9=\begin{array}{l}\text{median time between the date of patients' registration for a diagnostic examination }\\\text{and obtaining the results}\end{array}$$
**F_10 SCU**	Treatment	Rate of involved nodes after lymphadenectomy
		$$\text{F}_{10}=\frac{\text{number of metastatic lymphnodes removed during lymphadenectomy}}{\text{number of lymphnodes removed during lymphadenectomy}}$$
**F_11 SCU**	Diagnosis and screening	Staging according to the Breslow scale for thickness of the infiltration
		$$\text{F}_{11}=\frac{\text{number of patients with <0.8mm/0.8-1.0mm/1.0-2.0mm/2.0-4.0mm >4.0mm primary tumor}}{\text{number of patients}}$$

The clinical data from patients enrolled in the study were analyzed retrospectively from the start of the pilot study (May 1, 2020) to December 31, 2021. The dates of deaths were updated on March 31, 2022. The start of participation was determined by the date of the first visit, during which information about the diagnosis of melanoma was provided to patients based on HP examination. This date was also a reference point for the presented measures and moment in which each patient had to give their consent to participate in the pilot program and publication of its results. Data of patients who did not meet these criteria were not collected.

In total, 352 patients diagnosed with melanoma or melanoma in situ (ICD 10 - C43 and D03) in the SCU at LSOPHC for whom surgery was the primary treatment were included in the pilot study. Eleven patients who met the criteria for inclusion in the analysis did not agree to participate in the pilot study, however, they were covered by standard surgical care in our center. During the pilot study, there were 45 patients with unresectable or disseminated melanoma without previous surgical interventions in LSOPHC who underwent chemotherapy.

In this study, we compared the percentage of timely settled diagnostics between the 20 months of the pilot program and the similar period (January 1, 2019 to April 30, 2020) of creating the assumptions preceding it. These data come from 231 patients who underwent in-depth diagnostics (of whom 61 patients were also diagnosed initially in LSOPHC). The inclusion criteria were exactly the same as for patients later enrolled in the pilot program. During the period preceding the introduction of the pilot study, 61 patients were treated conservatively, all of them underwent at least one cycle of chemotherapy, and 4 of them also underwent radiotherapy.

## Results

**Fig. 1:**
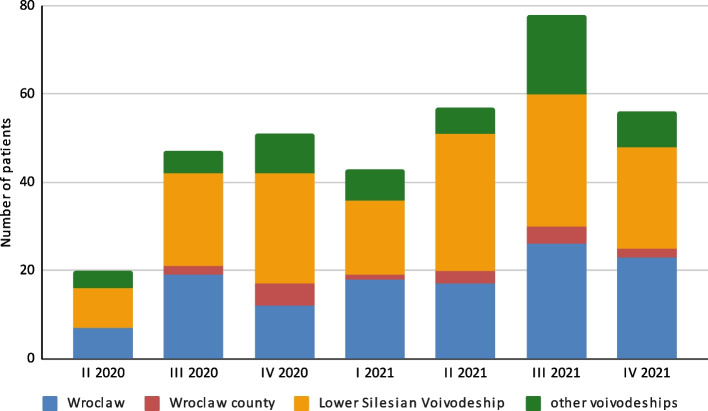
The number of patients by origin broken down into yearly quarters over the 20-month study period

Out of 352 patients enrolled in the pilot program, there were 171 women (48.6%) and 181 men (51.4%). The median ages for the women and men were 60.0 and 61.5 years, respectively (60.8 years for the whole group). 122 patients (34.7%) were from Wroclaw and 17 (4.8%) were from the rest of Wrocław county (Fig. 1). The largest group, consisting of 156 patients (44.3%), were from other areas of the Lower Silesian Voivodeship. 57 patients (16.2%) originated from 8 of the other 15 Polish voivodeships.

**Fig. 2:**
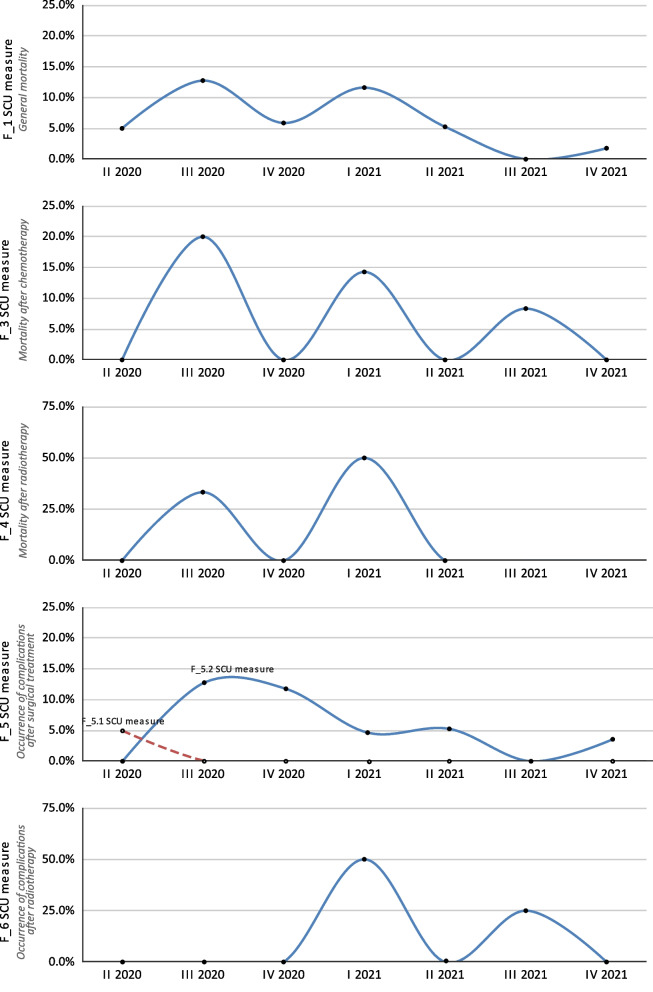
Results for the SCU F_1–F_6 measures (mortality following treatment and complications) broken down into yearly quarters over the 20-month study period

The F_1 SCU measure assessed the percentage of deaths within one year of the melanoma diagnosis. In the whole study group, the F_1 SCU measure was 5.4%. In the subgroups divided according to melanoma staging, the percentages increased with the advancement, and at stages I, II, III and IV were 0.6%, 3.7%, 10.9%, and 42.1%, respectively. The F_2 SCU measure evaluated the percentage of deaths within 30 days from surgery; however, no deaths occurred during this period. The other measures related to deaths were F_3 SCU and F_4 SCU, which assessed the percentage of patient deaths after the last cycles of chemotherapy or palliative radiotherapy, respectively. Of the patients enrolled in this pilot study, 94 patients underwent chemotherapy and the F_3 SCU measure was 6.4% (6 deaths). 2 out of the 17 patients who underwent radiotherapy died before end of the study; hence, the F_4 SCU measure was 11.8%. One patient required sudden hospitalization during the 30-day postoperative period (F_5.1 SCU measure: 0.4%) and 11 patients required additional consultation or outpatient treatment during that time (F_5.2 SCU measure: 8.2%). The F_6 SCU measure, which determined the percentage of patients who had complications after radiotherapy, was 11.8% (2 events among the 17 patients who received such treatment). The quarterly results for these measures are shown in Fig. 2.

**Fig. 3:**
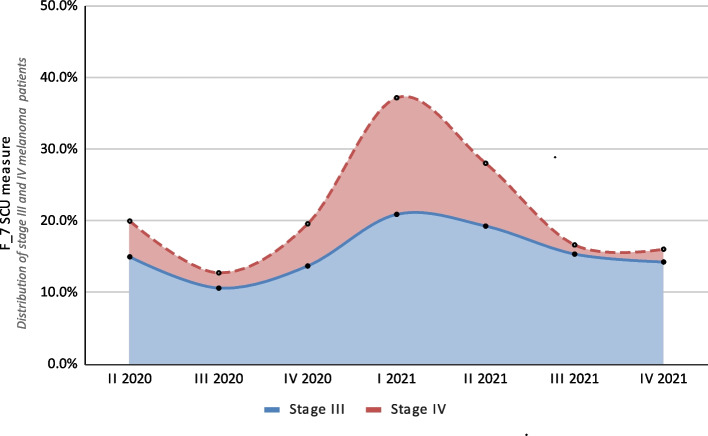
Results for the SCU F_7 measure (distribution of stage III and stage IV melanoma patients) broken down into yearly quarters over the 20-month study period

The F_7 SCU measure, which determined the percentage of patients with stages III and IV disease, was 15.6% (*n* = 55) and 5.4% (*n* = 19), respectively. The range of values observed in the subsequent yearly quarters varied from 1.3 to 16.3% for stage III, and from 10.6 to 20.9% for stage IV. Particularly high values (upper ranges) were observed in the first and second quarters of 2021, as shown in Fig. 3.

The completeness of the diagnostics for patients with melanoma was assessed by taking into account where it was originally done. Initial diagnostics were performed in 47 (13.4%) cases at the LSOPHC, and in 305 (86.6%) cases at external sites. In contrast, 327 patients (92.9%) started in-depth diagnostics at the LSOPHC, and 19 patients (5.4%) at external sites. In 6 patients, in-depth diagnostics were not performed.

**Fig. 4:**
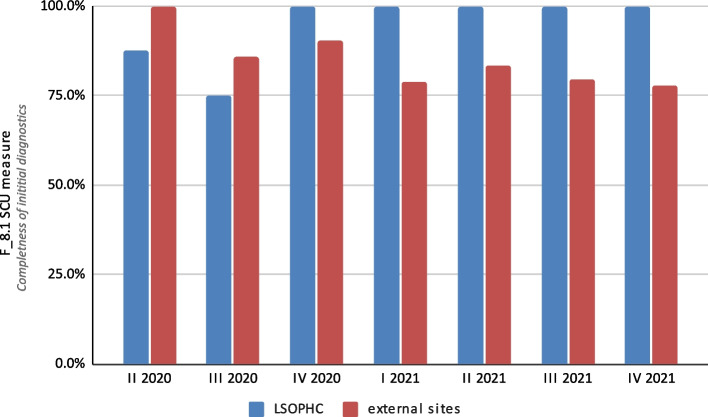
Results for the SCU F_8.1 measure (completeness of initial diagnostics) at the Lower Silesian Oncology, Pulmonology and Hematology Center (LSOPHC) and external sites broken down into yearly quarters over the 20-month study period

**Fig. 5:**
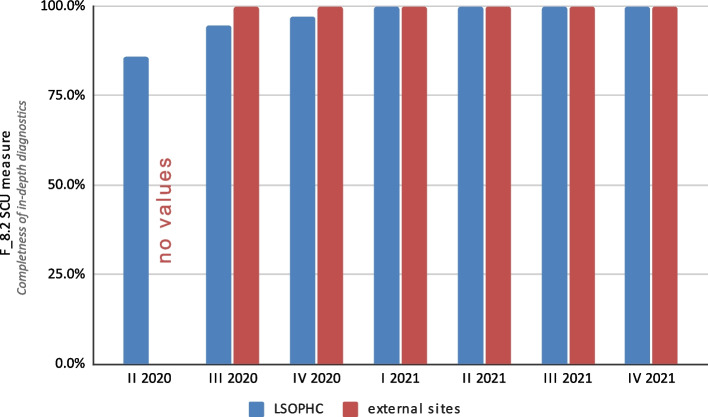
Results for the SCU F_8.2 measure (completeness of in-depth diagnostics) at the Lower Silesian Oncology, Pulmonology and Hematology Center (LSOPHC) and external sites broken down into yearly quarters over the 20-month study period

The F_8.1 SCU measure for initial diagnostic completeness was 90.3%, and the F_8.2 SCU measure for in-depth diagnostic completeness was 98.6%. Differences were noticed between the diagnostics performed at the LSOPHC and those performed at other sites (Fig. 4). The F_8.1 SCU measures for the LSOPHC and external units were 95.7% and 89.5%, respectively. For in-depth diagnostic completeness (F_8.2 SCU measure), it was 98.5% for cases diagnosed at the LSOPHC. At external sites, there were no deficiencies in the imaging examinations (Fig. 5).

Out of the 352 patients studied, incomplete diagnostics were noted in 44 cases (12.5%), of which 39 (11.1%) concerned pathomorphological examinations and 5 (1.4%) concerned imaging examinations. Microsatellitosis was not assessed in 32 patients. In 4 cases immunohistochemistry was not assessed, and in 3 cases key information about tumor thickness was missing. For in-depth diagnostics, 2 patients did not undergo an USG, and CT was not performed in 2 patients.

**Fig. 6:**
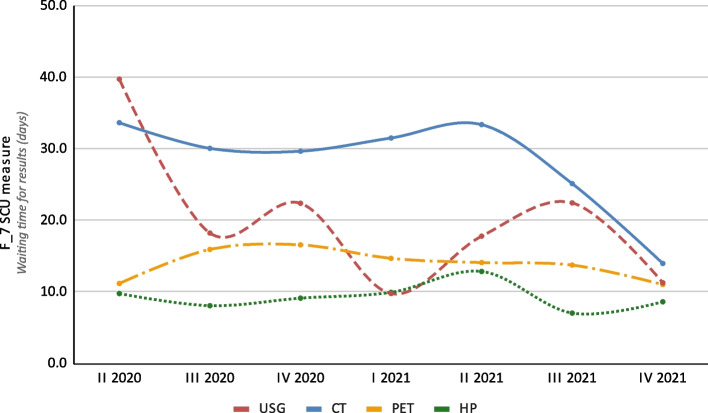
Results for the F_9 SCU measure (waiting time for results) for ultrasonography (USG), computed tomography (CT), positron emission tomography (PET), and histopathological (HP) examinations broken down into yearly quarters over the 20-month study period

During the study, 453 USG, 455 CT, 52 PET and 215 HP examinations were performed. The F_9 SCU measure results for USG, CT, PET and HP were 8.0 days, 18.0 days, 13.2 days, and 8.0 days, respectively. The results for this measure are shown in Fig. 6.

**Fig. 7:**
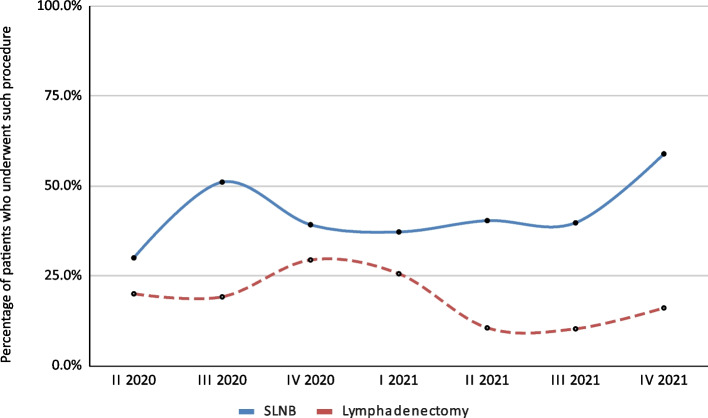
Quarterly trends in the percentage of patients undergoing sentinel node biopsy (SLNB) or lymphadenectomy

**Fig. 8:**
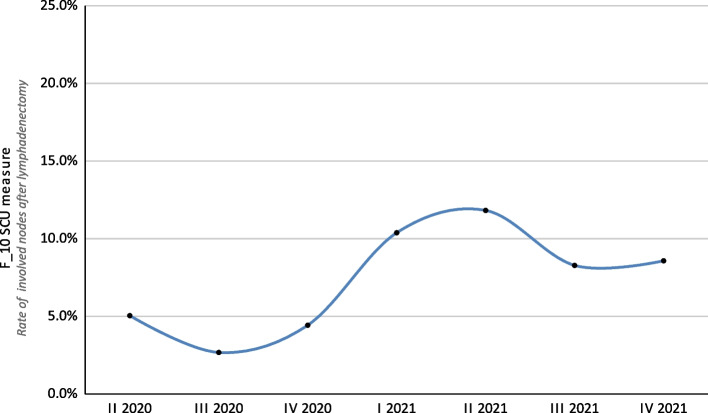
Results for the SCU F_11 measure (rate of involved nodes after lymphadenectomy) broken down into yearly quarters over the 20-month study period

In the analyzed group, lymphadenectomy was performed in 62 patients (17.6%). Of note, 153 patients (43.5%) underwent sentinel node biopsy (SNLB), and the trend for the percentage of patients who underwent SLNB and lymphadenectomy is presented in Fig. 7. The overall F_10 SCU score was 7.8%. A gradual increase in the score was observed over successive quarters. In 2020 it was between 2.7% and 5.1%, and in 2021 it was between 8.3% and 11.8% (Fig. 8).

The overall SCU F_10 measure, which assessed the percentage of patients by tumor thickness in accordance with the Breslow scale, was 25.6% for tumors thinner than 0.8 mm, 12.2% for those between 0.8 and 1.0 mm, 14.2% for those between 1.0 and 2.0 mm, 15.6% for those between 2.0 and 4.0 mm, and 22.7% for thicker than 4.0 mm. The SCU F_10 measure values did not reach 100% because 9.7% of patients with melanoma were diagnosed as Tis or Tx.

**Fig. 9:**
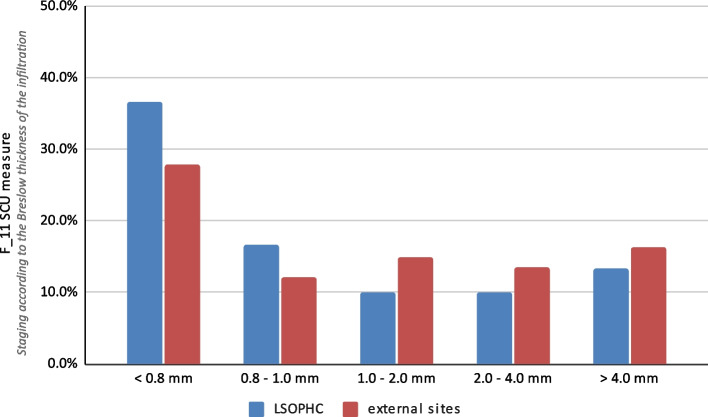
Comparison of the SCU F_10 measure scores (staging according to the Breslow thickness of infiltration) obtained at the Lower Silesian Oncology, Pulmonology and Hematology Center (LSOPHC) and at external sites

The primary tumor thickness in patients initially diagnosed at the LSOPHC differed from those diagnosed at external sites (Fig. 9). The SCU F_10 measure for tumors thinner than 0.8 mm was 40.5% for patients diagnosed initially at the LSOPHC and 23.5% for patients first diagnosed at external sites. In the LSOPHC subgroup, for tumors between 0.8 and 1.0 mm, 1.0–2.0 mm, 1.0–4.0 mm, and above 4.0 mm the SCU F_10 scores were 21.4%, 9.5%, 9.5%, and 9.5%, respectively. Whereas, among patients diagnosed externally, the SCU F_10 scores for the above-mentioned stages were 11.0%, 14.8%, 16.5% and 24.5%, respectively. These results are shown in Fig. 9.

**Fig. 10:**
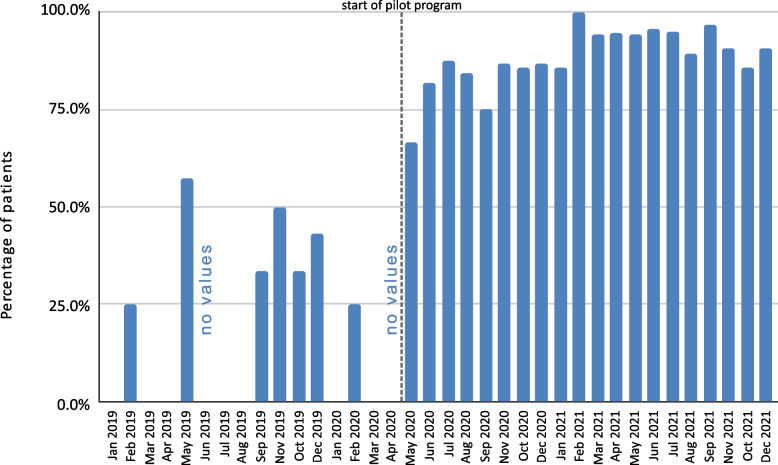
Maintenance of the timeliness of initial diagnostics before and after implementation of the pilot program

**Fig. 11:**
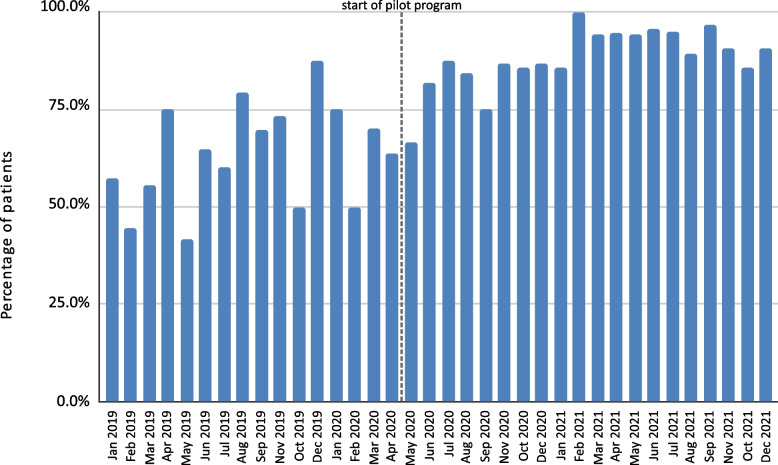
Maintenance of the timeliness of in-depth diagnostics before and after implementation of the pilot program

During the program, maintenance of the 3-week period for initial diagnostics in accordance with the DILO system was achieved in 87.8% of cases (Fig. 10), and for in-depth diagnostics it was achieved in 89.5% of cases (Fig. 11). In the period between the beginning of 2019 and the introduction of the pilot program, these results were 36.1% and 67.5%, respectively. A slightly positive trend was also observed for the initial and in-depth diagnostics during the pilot program itself.

## Discussion

The pilot programs for melanoma, described in the literature, mainly concern screening methods [17, 18]. The main reason for emphasizing this aspect seems to be primarily the high number of new cases observed every year [19]. Another frequently raised issue is the monitoring of treatment results, survival and quality of life among melanoma patients [20, 21]. To the best of our knowledge, there is a lack of evidence for using periodic audits and measures to monitor aspects presented in this article among melanoma patients treated in individual sites.

In recent years, much attention has been paid to the methods used for steadily monitoring and assessing clinical outcomes, which was implemented in the NCNPP and the melanoma pilot program presented in the current study [6, 7, 22]. A specific example, which was the basis for the creation of similar measures for melanoma patients, is presented in 2010 by the European Society of Breast Cancer Specialist set of benchmark quality indicators, which can be easily adapted to auditing and quality assurance in breast cancer centers [23]. Currently, in European countries, measures determining among others the completeness of diagnostics, waiting times or appropriate surgical approach are used also to increase the evaluation of the compliance and results of recommended treatment [24, 25].

Complications and deaths are the basic endpoints assessed in oncological treatment; therefore, 6 measures related to these issues were constructed. The developed measures allowed for sensitive monitoring, capturing even single events. However, a long-term analysis of their usability and trends (especially in patients from the last analyzed months) is necessary due to the fact that the reference point for the measures was mostly the date of commencement of participation in the project, and not at the start of individual treatment. It should also be mentioned that, due to the fact that the study group consisted of patients mainly under surgical care, the results may be potentially lower than reported, as the patients for whom surgery was not their primary treatment were not analyzed.

The evaluation of disease advancement in patients starting treatment is a valuable parameter for indirect assessment of the quality of diagnosis and early detection [26]. This study demonstrates the usefulness of the SCU F_7 and the SCU F_11 measures in that regard. An observably higher percentage of patients in the advanced stages of melanoma was reported in the first and second quarters of 2021 using the SCU F_7 measure. A potential explanation for this effect could be the limited access to healthcare services due to the Coronavirus Disease 2019 (COVID-19) pandemic and the patients’ fear of staying in medical facilities during this period [27, 28]. In general, the results obtained through the SCU F_7 measure provides insight into the current state of the patients’ disease advancement treated in a specific area [7]. From the perspective of the country where melanoma is diagnosed relatively late, it seems to be particularly important to assess the quality of early detection and screening effectiveness [29]. In this study, the SCU F_11 measure was also used to retrospectively assess the percentage of patients on each Breslow grade initially diagnosed at the LSOPHC and at external sites. It was shown that the patients initially diagnosed in the LSOPHC were diagnosed at earlier stages of the disease, which has an impact on prognosis.

In the course of this study, it was possible to assess the completeness of the HP examinations depending on location, which was measured using the SCU F_8 measure. Moreover, using the previously prepared checklists, it was possible to easily identify events related to deficiencies in the performed diagnostic tests and eliminate them in protocols from LSOPHC. The completeness of the pathomorphological protocol is of particular importance in therapeutic decision-making. Microsatellitosis, which was the most frequently unreported parameter at the beginning of the analyzed period, apart from being a marker of unfavorable tumor biology, determines the staging of disease (Stage III pN1c) and may change the therapeutic approach [30].

In the period covered by the analysis, an increase in the value of the SCU F_10 measure was demonstrated and the trend for moving away from lymphadenectomy to SLNB was evident. This is in line with the findings of the Multicenter Selective Lymphadenectomy Trial I (MSLT-I), the MSLT-II, and the German Dermatologic Cooperative Oncology Group-Selective Lymphadenectomy Trial (DeCOG-SLT) that were reported recently [31]. An active surveillance strategy among SLNB positive melanoma patients is gaining importance, due the possibility of avoiding unnecessary, more invasive surgical treatments and potential complications [32]. However, currently, data on the effects of this procedure on long-term overall survival remains limited [33].

The analysis of the SCU F_9 measure allowed for identification of the weak points in the diagnostics carried out at the LSOPHC. There were no reductions in the F_9 SCU measure broken down into yearly quarters for HP examination (range: 7.0 days − 12.9 days) and PET (11.0 days − 16.6 days); however, these were in the minority and their duration was significantly lower than 3–4 weeks predicted for initial and in-depth diagnostics. An important shortening of the waiting times for the results of USG (39.8 days − 18.2 days in the first two observed yearly quarters and 22.5–11.3 in the last two) and CT (33.7 days − 30.1 days in the first two observed yearly quarters and 25.1 days − 14.0 days in the last two),, was observed. The main reason for this was the introduction of medical coordinators who took an active part in the scheduling examinations. Due to the fact that USG and CT are key examinations in the diagnosis of melanoma (accounted for 77.3% of the examinations performed in the study group), increased attention was placed on these procedures by all staff during the pilot program. The main benefits of the shortened diagnosis time, reported also in the NCNPP, were earlier treatment initiation, which could influence the clinical outcome, and increased satisfaction with the care provided [7].

## Conclusion

The current data allowed us to verify the usefulness of the introduced measures as feasible tools for insight into the selected clinical issues. It was also shown that the measures allowed for a multidimensional analysis of the quality of treatment and diagnostics. Furthermore, the developed measures may be a valuable tool for constant monitoring, and identifying and addressing key factors influencing diagnostic and treatment outcomes. Due to the significant impact on making therapeutic decisions, and thus the possible clinical outcome, the authors perceive the special role of introducing the SCU F_8 measure, which allowed for the verification of the completeness of the pathomorphological protocol. Introduction of the solutions proposed in this pilot study improved the quality and shortened the duration of diagnostics. Shortening the time for initial and in-depth diagnostics was possible due to the introduction of medical coordinators who were responsible for scheduling examinations and patients’ visits to the clinic.

## Data Availability

The datasets used and analyzed during the current study are available from the corresponding author on reasonable request.

## Abbreviations

COVID-19: Coronavirus Disease 2019
CT: Computed tomography
DILO system: Pol. system Diagnostyki i Leczenia Onkologicznego; eng. Diagnostic and Oncological Treatment system
HP: Histopathological protocol
LSOPHC: Lower Silesian Oncology, Pulmonology and Hematology Center
NCNPP: National Cancer Network’s Pilot Program
PET: Positron emission tomography
SCU: Skin Cancer Unit
USG: Ultrasonography
